# Left atrial venoarterial extracorporeal membrane oxygenation as a bridge to surgical aortic valve replacement in cardiogenic shock

**DOI:** 10.1016/j.xjtc.2026.102309

**Published:** 2026-03-20

**Authors:** Shaan Asif, Ivan Hanson, Alessandro Vivacqua, Jeffrey Altshuler, Thomas Schwann, Rakesh M. Suri, Ahmad Jabri, Bogdan Kindzelski

**Affiliations:** a Department of Cardiovascular Surgery, Corewell Health William Beaumont University Hospital, Royal Oak, Mich; b Oakland University William Beaumont School of Medicine, Rochester, Mich; c Department of Cardiovascular Medicine, Corewell Health William Beaumont University Hospital, Royal Oak, Mich

**Figure:**
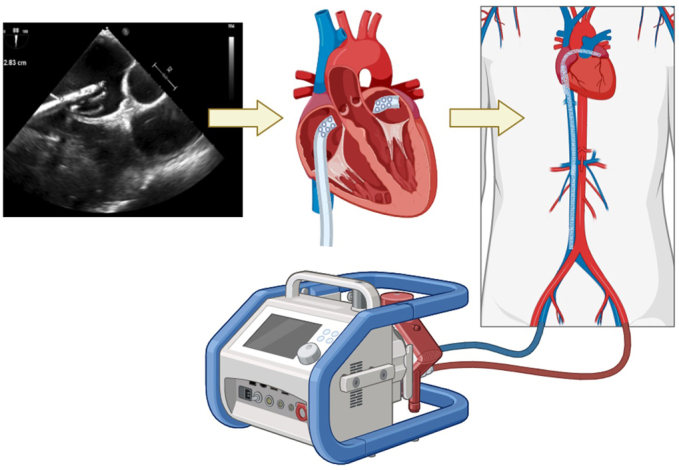
Left atrial venoarterial ECMO with transesophageal image depicting cannula position.

**Central Message**

In the case of aortic insufficiency resulting in cardiogenic shock, left atrial venoarterial ECMO results in left ventricular unloading, improved perfusion, and a viable bridge-to-surgery pathway.

Valvular cardiogenic shock results in impaired cardiac output and/or end-organ hypoperfusion despite there being an adequate preload to the heart.^1^ Although venoarterial extracorporeal membrane oxygenation (VA-ECMO) augments systemic perfusion, it also raises left ventricular (LV) afterload through retrograde aortic flow when peripheral VA-ECMO is used. This can lead to LV distension, elevated LV end-diastolic pressure, increased myocardial oxygen consumption, and pulmonary edema.^1^, ^2^, ^3^ Left atrial venoarterial ECMO (LAVA-ECMO), a modification of conventional VA-ECMO, is intended to overcome the problem of LV distension. Inflow into the LAVA-ECMO circuit is secured from the left and right atria through a transseptal cannula with multiple side holes drawing from both sides of the inter-atrial septum, thereby achieving passive biventricular unloading while optimizing end organ perfusion. We present a case of LAVA-ECMO used as a bridge to definitive surgery in an acutely decompensated and critically ill patient with severe aortic insufficiency (AI) and review the relevant literature.

## Case Study

**Figure 1:**
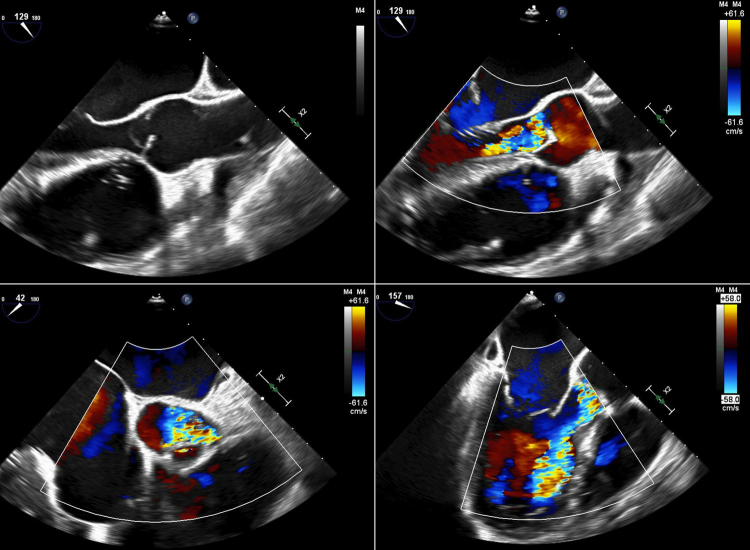
Representative transesophageal echo images showing severe aortic insufficiency with prolapse of the noncoronary cusp and malcoaptation of the right and left coronary cusps.

**Figure 2:**
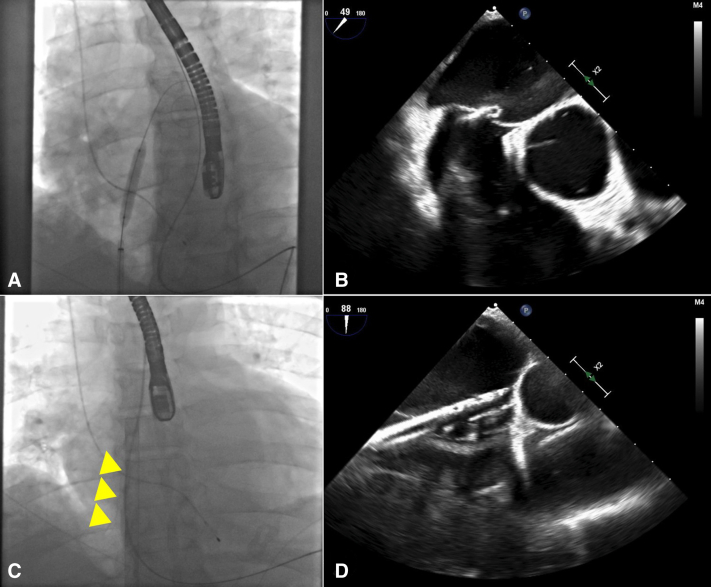
Steps for left atrial venous cannula placement. Femoral venous access is obtained and an 8-French Baylis transseptal system is used with TEE guidance for transeptal access. A, A wire is placed in the left upper pulmonary vein and an atrial septostomy done using an 8- × 40-mm balloon. B, TEE showing transseptal access and balloon inflation. C, 25-French ECMO cannula with multiple fenestrations at the distal end is advanced. D, TEE showing the distal end of the cannula positioned 3 cm across the interatrial septum for drainage from both atria. *TEE*, Transesophageal echocardiography; *ECMO*, extracorporeal membrane oxygenation.

The patient provided informed consent for the deidentified publication of the details of the clinical scenario. Institutional review board approval was not required. A 65-year-old man with a medical history of hypertension, chronic heart failure with preserved ejection fraction, chronic obstructive pulmonary disease with active tobacco abuse, and obesity presented with acute heart failure exacerbation. He was treated medically with diuresis and left against medical advice before full workup. Four days later, he presented to the emergency department with worsening respiratory distress, hypotension, and multiorgan dysfunction. His lactate peaked at 15.8 mmol/L, he had evidence of shock liver (alanine transaminase 4666 U/L and alanine aminotransferase 1496 U/L), acute renal failure (creatinine 5.36 mg/dL), and metabolic acidosis. He was started on inotropic support and a cardiac shock team was activated. After a multidisciplinary discussion, the patient was at prohibitive risk for emergent surgery or off-label transcatheter aortic valve replacement. He was temporized on LAVA-ECMO as a bridge to definitive aortic valve replacement. The patient was brought to the hybrid operating room. Transesophageal echocardiography revealed severe AI (Figure 1). Femoral arterial and venous access was obtained. Heparin was administered with a target activated clotting time >300, and a 20-French arterial cannula (Fem-Flex, Edwards Lifesciences) was placed in the femoral artery. Next, for the venous cannulation, a transseptal puncture was performed (Figure 2). Atrial septostomy was performed and a 25-French venous drainage cannula with multiple fenestrations (Bio-Medicus; Medtronic) was advanced about 3 cm across the interatrial septum for drainage from both the left and right atria. The cannula was secured with multiple sutures to prevent dislodgement. After the initiation of LAVA-ECMO (Cardiohelp System; Getinge), systemic perfusion and acid–base status improved markedly. Over the course of the next day, his lactate levels normalized, kidney function improved, and shock liver stabilized. An echocardiogram was performed to confirm cannula position and no worsening LV distension, and the patient was sedated and kept paralyzed to mitigate cannula dislodgement.

He was taken to the operating room next day for definitive aortic valve replacement. A cutdown was made on the left groin and the femoral vessels were exposed. Heparin was administered; the femoral venous cannula was retracted from the left atrium and guided into superior vena cava. Next, the tubing for the LAVA ECMO circuit was converted for cardiopulmonary bypass and connected to the already placed left femoral arterial cannula and right femoral venous cannula. Cardiopulmonary bypass was commenced, the heart was arrested and, on inspection of his valve, there appeared to be torn segments of the noncoronary cusp with evidence of healed endocarditis. An aortic valve replacement was performed with a 27-mm tissue valve. Next the cava was snared, right atrium was opened, and the atrial septal defect closed primarily. Postoperative transesophageal echocardiography showed well-functioning bioprosthesis with no evidence of paravalvular leak or residual atrial septal defect.

Postoperatively, the patient was transferred to the cardiovascular intensive care unit and had an uneventful postoperative cardiovascular course. On 2-year follow up, his echocardiogram showed a left ventricular ejection fraction of 60% with normally functioning valve. On 3-year follow-up with his cardiologist, the patient continues to do well with stable heart function and no residual cardiac sequalae.

## Discussion

LAVA-ECMO provides simultaneous biatrial drainage, leading to marked reductions in LV and RV preload, end-diastolic pressure, pulmonary capillary wedge pressure, and pulmonary artery pressures.^4^ Collectively, these effects alleviate pulmonary congestion and decrease myocardial oxygen demand. However, these patients require frequent monitoring with a Swan-Ganz catheter (Edwards Lifesciences) to ensure adequate LV unloading. In a single-center analysis of 68 patients, LAVA-ECMO resulted in significant hemodynamic improvement within 24 hours of initiation, including mean reductions in right atrial pressure by 5 mm Hg, mean pulmonary artery pressure by 9 mm Hg, pulmonary capillary wedge pressure by 10 mm Hg, and LV end-diastolic pressure by 14 mm Hg.^4^ These physiologic benefits are advantageous in patients with severe mitral or aortic regurgitation, where effective LV unloading is essential to promote myocardial recovery.^2^^,^^4^ Furthermore, in patients who are in critical cardiogenic shock due to acute severe AI, options for mechanical support and venting strategies are limited, as the use of VA-ECMO in conjunction with a microaxial flow pump (Impella; Abiomed) can cause worsening valvular insufficiency and recirculation.^5^ Therefore, LAVA-ECMO is a viable option for acute severe AI in cardiogenic shock and should be part of the armamentarium for treatment in this patient subset. This is a promising mechanical circulatory support platform in selected patients, but its full role warrants further experience and study.

## Conflict of Interest Statement

The authors reported no conflicts of interest.

The *Journal* policy requires editors and reviewers to disclose conflicts of interest and to decline handling or reviewing manuscripts for which they may have a conflict of interest. The editors and reviewers of this article have no conflicts of interest.
